# Lung Cavities in Chronic Thromboembolic Pulmonary Hypertension

**DOI:** 10.6061/clinics/2020/e1373

**Published:** 2020-01-06

**Authors:** Caio Julio Cesar dos Santos Fernandes, Ellen Pierre de Oliveira, Willian Salibe-Filho, Mario Terra-Filho, Carlos Vianna Poyares Jardim, Luciana Tamie Kato-Morinaga, Susana Hoette, Rogerio de Souza

**Affiliations:** I Unidade de Hipertensao Pulmonar, Departamento de Pneumologia, Instituto do Coracao (InCor), Hospital das Clinicas HCFMUSP, Faculdade de Medicina, Universidade de Sao Paulo, Sao Paulo, SP, BR; II Instituto do Cancer do Estado de São Paulo (ICESP), Hospital das Clinicas HCFMUSP, Faculdade de Medicina, Universidade de Sao Paulo, Sao Paulo, SP, BR; III Hospital Sirio Libanes, Sao Paulo, SP, BR

**Keywords:** Chronic Thromboembolic Pulmonary Hypertension, Lung Cavities, Pulmonary Infarction, Chronic Granulomatous Diseases, Infection

## Abstract

**OBJECTIVES:**

Chronic thromboembolic pulmonary hypertension (CTEPH) is a unique form of pulmonary hypertension (PH) that arises from obstruction of the pulmonary vessels by recanalized thromboembolic material. CTEPH has a wide range of radiologic presentations. Commonly, it presents as main pulmonary artery enlargement, peripheral vascular obstructions, bronchial artery dilations, and mosaic attenuation patterns. Nevertheless, other uncommon presentations have been described, such as lung cavities. These lesions may be solely related to chronic lung parenchyma ischemia but may also be a consequence of concomitant chronic infectious conditions. The objective of this study was to evaluate the different etiologies that cause lung cavities in CTEPH patients.

**METHODS:**

A retrospective data analysis of the medical records of CTEPH patients in a single reference PH center that contained or mentioned lung cavities was conducted between 2013 and 2016.

**RESULTS:**

Seven CTEPH patients with lung cavities were identified. The cavities had different sizes, locations, and wall thicknesses. In two patients, the cavities were attributed to pulmonary infarction; in 5 patients, an infectious etiology was identified.

**CONCLUSION:**

Despite the possibility of being solely associated with chronic lung parenchyma ischemia, most cases of lung cavities in CTEPH patients were associated with chronic granulomatous diseases, reinforcing the need for active investigation of infectious agents in this setting.

## INTRODUCTION

Chronic thromboembolic pulmonary hypertension (CTEPH) is a unique form of pulmonary hypertension (PH) that arises from obstruction of the pulmonary vessels by organized thromboembolic material (1). CTEPH has historically been shown to be a challenging clinical entity that is frequently underdiagnosed and undertreated. The primary therapeutic approach that should be implemented for CTEPH is pulmonary endarterectomy (PEA), a surgery that removes the occluding material from the pulmonary vessels as well as the vascular endothelium (2), and lifelong anticoagulation (3).

CTEPH has a wide range of radiologic presentations. Unlike acute pulmonary embolism (4-6), CTEPH frequently presents as enlargement of the main pulmonary artery, peripheral vascular obstructions, bronchial artery dilations, and mosaic attenuation patterns (7). Nevertheless, other uncommon presentations have been described, such as lung cavities (8-11). These lesions may be solely related to chronic lung parenchyma ischemia but may also be a consequence of concomitant chronic infectious conditions and have significant management implications. Herein, this study describes seven CTEPH patients with lung cavities and discusses the diagnostic approach and management of these conditions.

## METHODS AND RESULTS

From 2013 to 2016, seven CTEPH patients presented lung cavities at diagnosis in the main reference center for pulmonary hypertension in Brazil (Heart Institute, SP), a country with high endemic rates of chronic granulomatous lung diseases. During this 4-year period, we evaluated 140 CTEPH patients at our center and performed PEA in approximately 110 patients. These CTEPH patients mostly presented with symptoms similar to those of other forms of PH, progressive dyspnea and symptoms of right ventricular failure, which was the most prevalent presentation.

All PH patients at our center followed the diagnostic algorithm suggested by European Society of Cardiology and European Respiratory Society and were reviewed in 2015 (12). Therefore, during the evaluation, all PH patients underwent a lung perfusion scan, the gold standard technique to CTEPH confirmation (13). Once CTEPH was identified, the patients were evaluated as possible candidates for PEA. However, PEA is a major surgery that includes extracorporeal perfusion support. Lung cavitations, in this setting, could affect the surgical outcome since chronic infections could be associated with these cavitations. Our decision was to perform PEA only when the etiology of the lung cavitation in CTEPH was clarified.

All CTEPH patients who presented lung cavitations (two females and five males) had dyspnea at the first medical appointment; four were considered New York Heart Association functional class (FC II), two were FC III, and one was FC IV. Three patients did not present with acute PE prior to the CTEPH diagnosis. Two patients had a background of acute PE, and the two remaining patients had acute PE associated with deep vein thrombosis (DVT). Regarding their previous medical history, one patient had chronic obstructive pulmonary disease (COPD), and another had Proteus syndrome (a rare congenital disorder that causes skin overgrowth and atypical bone development, often accompanied by tumors over half the body).

All patients underwent transthoracic echocardiography that showed a pulmonary artery systolic pressure (PASP) greater than 40 mmHg. To characterize pulmonary hypertension, the patients underwent right heart catheterization. The clinical and hemodynamic data are described in Table 1.

**Figure 1:**
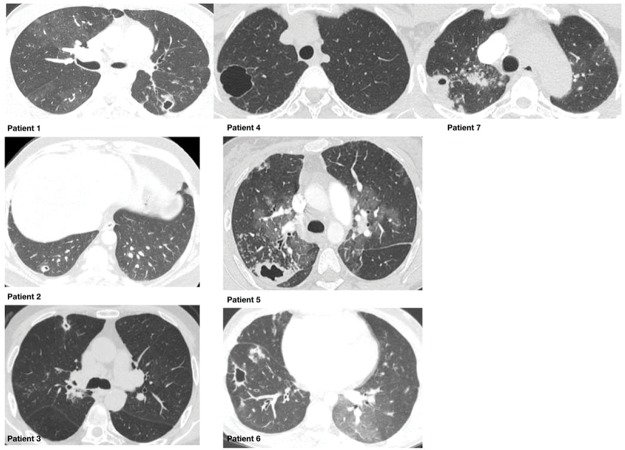
Chest CT scans of 7 CTEPH patients with lung cavities. Aspects of the cavity such as wall thickness were not able to distinguish infectious diseases from ischaemic cavities.

The CTEPH diagnosis was made after three months of adequate therapeutic anticoagulation. All patients had segmental perfusion defects, which were identified by a nuclear lung perfusion scan. Thoracic computed tomography angiography (MCTA) showed pulmonary artery obstructions in all patients. All presented lung cavities were of different sizes and thicknesses, were surrounded by ground-glass opacities, and were concordant with the pulmonary artery obstruction regions. The diameter of the cavities varied from 9.4 to 25 mm, with all but one presenting a wall thickness greater than 2 mm (Figure 1).

The diagnostic procedure and resulting diagnosis for each patient are described in Table 2. All but one patient with chronic infectious diseases were successfully treated and underwent pulmonary endarterectomy. Patients 4 and 5 died of right ventricular failure, and the appropriate diagnosis was only made at necropsy. No infectious agent was identified for patient 4. Patient 6 underwent a bronchoalveolar lavage, but no microorganisms were detected, and the lesion was considered purely associated with the pulmonary infarction.

## DISCUSSION

The study results demonstrate that, despite the possibility of being solely associated with chronic lung parenchyma ischemia, most cases of lung cavities in CTEPH were associated with chronic granulomatous diseases, reinforcing the need for active investigation of infectious agents in this setting.

CTEPH has a variety of radiologic presentations. The most common signs are vascular filling defects, abrupt vessel cut off, and pulmonary parenchyma mosaic attenuation. Eventually, there are also findings associated with lung infarction, such as parenchymal bands, wedge-shaped densities, and cavities (14). Nevertheless, since the lung has dual perfusion sources (bronchial and pulmonary circulation), even severe vascular obstructions present in CTEPH rarely lead to advanced necrosis and lung cavities, given that the bronchial circulation provides some degree of perfusion. In some clinical situations, however, the vascular balance may be disturbed, such as in the presence of infections. Infectious diseases increase the basal metabolic demand systemically and locally. In this setting, the blood supply provided exclusively by the bronchial circulation may not be enough, and lung necrosis may occur, and a cavity may develop as a consequence.

Lung cavities are a result of several distinct pathological processes, such as cancer (15), suppurative necrosis (pyogenic lung abscess), caseous necrosis (tuberculosis) or cystic dilation of the lung structures (16). The radiographic appearance of a cavity lesion can sometimes be useful to differentiate among a broad spectrum of etiologies but should be combined with clinical and laboratory data for an accurate diagnosis. One traditional method for classifying excavated lung lesions is the use of the cavity wall thickness. Infectious diseases usually present thick walls and may be surrounded by micronodules on chest CT and thus, these findings frequently favor the possibility of infection. Nevertheless, in this case series, cavity wall thickness was not able to distinguish infectious diseases from ischaemic cavities.

One critical condition classically associated with pulmonary cavity disease is *Mycobacterium tuberculosis* (16). Due to its high prevalence, the awareness of lung tuberculosis should be high, mainly in regions with high endemic levels for the disease. In fact, despite other potential causes for the lung cavity, such as a vascular perfusion deficit, the majority of these patients had some form of mycobacteria infection as the main cause of the cavity. The adequate identification of the etiology of the lung cavity in CTEPH may lead to a specific anti-infective therapy, when necessary, and have a direct impact on the outcome of such a severe condition. Similarly, nontuberculous mycobacterial lung disease was also associated with newly formed lung cavitations in CTEPH patients (17,18).

## CONCLUSIONS

Lung cavities are a possible CT finding of CTEPH patients. These cavities may be due solely to vascular impairment. Nevertheless, an adequate evaluation of the cavity etiology is mandatory. In most CTEPH patients with lung cavitations, it is possible to identify an infectious agent, which may warrant a specific anti-infective therapy approach and impact the outcomes of severe conditions such as CTEPH.

## AUTHOR CONTRIBUTIONS

Fernandes CJCS wrote the manuscript final drafting. Oliveira EP, Salibe-Filho W, Terra-Filho M, Kato-Morinaga LT and Hoette S were responsible for data acquisition. Jardim CVP was responsible for the management and review of the manuscript final drafting. Souza R reviewed the manuscript final drafting.

## Figures and Tables

**Table 1:** Baseline data of CTEPH patients with lung cavities.

Patients	1	2	3	4	5	6	7
Age (years)	26	42	65	28	37	24	64
Sex	Male	Male	Male	Female	Female	Male	Male
Medical History			COPD		Proteus syndrome		
Embolism History	PE+DVT	PE			PE	PE+DVT	
Functional Class	II	III	II	IV	II	III	II
Echocardiogram PSAP (mmHg)	60	82	69	94	112	96	70
TC6M (m)	541	214		360	262	460	
BNP (pg/mL)	72	187	514	246	367	180	639
mPAP (mmHg)	63	52	42	53	64	39	53
PCWP (mmHg)	9	11	18	10	14	11	19
Cardiac Output (L/min)	5.5	4.8	4.5	3.2	3.1	4.1	2.7
Pulmonary Vascular Resistance (WOOD)	9.8	8.5	5.3	13.7	16.1	5.7	12.5

**Table 2:** Diagnosis of the cavities.

Patient	Procedure	Diagnosis
1	Open lung biopsy	Mycobacterium tuberculosis
2	Open lung biopsy	Mycobacterium interjectum
3	Bronchoalveolar lavage	Aspergillus sp
4	Necropsy	Pulmonary Infarction
5	Necropsy	Mycobacterium tuberculosis
6	Bronchoalveolar lavage	Pulmonary Infarction
7	Bronchoalveolar lavage	Mycobacterium tuberculosis
